# Single-Dose Pharmacokinetics and Preliminary Safety Assessment with Use of CBD-Rich Hemp Nutraceutical in Healthy Dogs and Cats

**DOI:** 10.3390/ani9100832

**Published:** 2019-10-19

**Authors:** Kelly A. Deabold, Wayne S. Schwark, Lisa Wolf, Joseph J. Wakshlag

**Affiliations:** 1 Department of Comparative Diagnostic Population Medicine, University of Florida College of Veterinary Medicine, Gainesville, FL 32608, USA; kdeabold@icloud.com; 2 Department of Molecular Medicine, Cornell College of Veterinary Medicine, Ithaca, NY 14853, USA; wss2@cornell.edu; 3 Proteomics and Metabolomics Facility, Colorado State University, Fort Collins, CO 80521, USA; Lisa.Wolfe@colostate.edu; 4 Department of Clinical Sciences, Cornell College of Veterinary Medicine, Ithaca, NY 14853, USA

**Keywords:** hemp, cannabidiol, dog, cat, pharmacokinetics, toxicity

## Abstract

**Simple Summary:**

The use of cannabidiol (CBD)-rich hemp-based nutraceuticals is increasing in dogs and cats for disorders related to anxiety, seizures, cancer and pain. To date, there is little information related to appropriate dosing or long-term effects on serum chemistry or complete blood counts (CBC), and little data on the pharmacokinetics of single- or long-term dosing in dogs and cats. Single-dose pharmacokinetics and preliminary 12-week serum chemistry and complete blood counts are reported here showing short pharmacokinetic half-lives of cannabidiol in dogs and cats, with cats showing far lower oral absorption kinetics or rapid elimination suggesting dosing may differ between the two species. Fortunately, there were no changes in physical examination and few changes in the CBC and serum chemistry parameters suggesting the relative safety of oral supplementation over 12 weeks. One of the eight cats displayed a persistent rise in the serum alanine amino transferase (ALT) enzyme outside of the reference range and cats commonly displayed excessive licking and head shaking with administration of the oil. Based on these and other recent data, CBD-rich hemp nutraceuticals appear to be safe in healthy adult dogs, while more work in cats is needed to fully understand utility and absorption.

**Abstract:**

The use of CBD-rich hemp products is becoming popular among pet owners with no long-term safety data related to consumption in adult dogs and cats. The purpose of this study was to determine the single-dose oral pharmacokinetics of CBD, and to provide a preliminary assessment of safety and adverse effects during 12-week administration using a hemp-based product in healthy dogs and cats. Eight of each species were provided a 2 mg/kg total CBD concentration orally twice daily for 12 weeks with screening of single-dose pharmacokinetics in six of each species. Pharmacokinetics revealed a mean maximum concentration (Cmax) of 301 ng/mL and 43 ng/mL, area under the curve (AUC) of 1297 ng-h/mL and 164 ng-h/mL, and time to maximal concentration (Tmax) of 1.4 h and 2 h, for dogs and cats, respectively. Serum chemistry and CBC results showed no clinically significant alterations, however one cat showed a persistent rise in alanine aminotransferase (ALT) above the reference range for the duration of the trial. In healthy dogs and cats, an oral CBD-rich hemp supplement administered every 12 h was not detrimental based on CBC or biochemistry values. Cats do appear to absorb or eliminate CBD differently than dogs, showing lower serum concentrations and adverse effects of excessive licking and head-shaking during oil administration.

## 1. Introduction

Cannabidiol (CBD) use is becoming increasingly popular in both human and veterinary medicine due to federal legislation changes for hemp, making distribution of hemp and hemp-based products legal in the United States, so long as they contain less than 0.3% tetrahydrocannabinol (THC) [1]. Veterinary consensus is that research is needed and many would consider its use, however current food and drug administration recommendations discourage the use of hemp products in pets, and regulatory policy surrounding hemp use is uncertain [2]. The *Cannabis sativa* and *indica* plants contain a multitude of chemicals including phytocannabinoids, terpenoids, flavonoids, and sterols [2,3,4,5,6]. The phytocannabinoid of abundance in hemp is CBD and its derived acid, cannabidiolic acid (CBDA) which is rapidly converted to CBD with heat or pH changes; with lesser amounts of THC, cannabigerol, cannabichromene and cannabinol.

THC is the main cannabinoid found in Cannabis marijuana. It is responsible for the psychotropic effects and the toxic effects of THC have been well documented [7,8]. Conversely, CBD is the main cannabioid found in hemp [9]. CBD has been suggested to have a range of pharmacologic actions, such as allosteric inhibitory properties on the CB1 receptor, and is non-psychotropic, highly tolerable, with no known clinical toxicity recorded [2,3,4,5,9]. Mechanistically, CBD appears to be a partial agonist for the CB2 receptor as well as an agonist of the transient receptor potential cation channels (TRPV), 5 hydroxytryptophan 5A receptor, glycine receptors, and an inhibitor of adenosine uptake activity at low micromolar to high nanomolar concentrations. These attributes, as well as the ability to stimulate transcription and translational activity via the peroxisome proliferation receptor, and ability to down-regulate cyclooxygenase expression and activity makes CBD an attractive cannabinoid for therapeutic use in companion animals [9,10]. More interestingly, some evidence points to the potential for whole plant-derived CBD being more effective than synthesized or highly purified CBD, suggesting that other cannabinoids or terpenes may have additive or synergistic effects with CBD [9].

It is through these non-canonical receptor actions that hemp-derived CBD has been proposed to have clinical utility in human medicine with the focus of hemp-derived CBD use in seizures, mental disorders, chronic pain and cancer quality of life [2,3,4,9]. There is limited evidence for treating these same disease processes in animals with recent pilot studies investigating seizure management and osteoarthritis pain, showing promising results [11,12]. Currently, the longest pharmacokinetic study in dogs has been 6 weeks using 10 and 20 mg/kg and the longest clinical study using 2.5 mg/kg whole plant-derived CBD twice daily for 12 weeks; with both of these studies showing elevations in alkaline phosphatase enzyme and large inter-individual variability in serum CBD concentrations [11,13].

Recent work suggests that CBD can be absorbed most efficiently in an oil base and undergoes rapid first-pass metabolism with oxidation, carboxylation, and glucuronidation leading to a variety of metabolites [13,14,15]. The metabolites formed and the metabolic pathways utilized appear to vary between species [16,17]. Oil-based products delivering between 2–10 mg/kg of CBD show average maximal serum concentrations reaching 100–600 ng/mL within two hours of treatment and half- elimination rates of approximately 4 h in dogs [12,18].

The purpose of this study was to determine the pharmacokinetics and preliminary safety of an oral canine whole-plant CBD-infused soft chew and oral feline CBD-infused fish oil. It was hypothesized that there would be no significant changes in complete blood count (CBC) or serum biochemistry values and that the only adverse effects observed would be associated with oral administration of the product, particularly in cats.

## 2. Materials and Methods

The protocols used in these studies were reviewed and approved prior to study initiation by the Summit Ridge Farms’ Institutional Animal Care and Use Committee and were in compliance with the Animal Welfare Act. Animals were housed in the proper facilities and cared for in accordance to the Animal Welfare Act (United States Department of Agriculture Registration, No. 23-R-0126).

### 2.1. Dogs

Eight fasted, healthy, purpose-bred research Beagle dogs with a mean age of 3.2 years ranging from 11 months to 5 years of age, weighing an average of 9.7 kg (7.4 to 12.0 kg), were included in the study. The dogs were offered ElleVet Mobility Chews (ElleVet Sciences; Portland, ME, USA) at a dose of 2 mg/kg twice daily for 84 days. Small chews contained 10 mg of CBD as a 50% mix of CBD (5 mg per chew) and CBDA (CBDA—5 mg per chew). Large soft chews containing approximately 15 mg of CBD (equal mix of CBD/CBDA) were also used in the study. When necessary, a combination of large and small chews were used or partitioned in half to reach the appropriate dose for 84 days. All dogs had been fasted from the prior day and were not fed until 8 h after the initial dosing.

Prior to the start of and every 4 weeks over the course of the study, 5 mm of blood was collected via jugular venipuncture in sterile syringes. Samples were split into two tubes, a red top coagulation tube and an ethylenediaminetetraacetic acid tube. Red top tubes were spun in a refrigerated centrifuge for 15 min at 1512× *g* after being allowed to clot for 10 min. Blood samples were packaged and sent priority-overnight for analysis to ANTECH Diagnostics (Fountain Valley, CA, USA). A white blood cell count (WBC), red blood cell (RBC) count, hemoglobin (Hb), hematocrit (HCT), Mean corpuscle volume (MCV), mean corpuscle hemoglobin concentration (MCHC), mean corpuscle hemoglobin (MCH), and platelet count along with a complete differential was performed. A serum chemistry screen was performed consisting of, albumin, alkaline phosphatase (ALP), alanine aminotransferase (ALT), aspartate aminotransferase (AST), calcium, chloride, cholesterol, creatinine, creatine kinase (CK), gamma glutamyl transferase (GGT), glucose, globulin, magnesium, phosphorus, potassium, sodium, total bilirubin, total protein, triglycerides, and urea nitrogen (BUN).

### 2.2. Cats

Eight fasted, healthy, purpose-bred domestic shorthair research cats with a mean age of 4.5 years ranging from 2–6.3 years of age, weighing an average of 4.2 kg (3.3 to 5.2 kg) were included in the study. The cats were dosed with CBD-infused fish oil (50/50% mix of CBD and CBDA; ElleVet Sciences; Portland, ME, USA) at 2 mg/kg. The total dose per 24 h period was 4 mg/kg, for 84 days. The initial pharmacokinetic dosing was done with capsules to ensure consumption and all cats were fasted from the previous day and were not fed until 6 h after initial dosing.

Prior to the start of and every 4 weeks throughout the course of the study, 5 mL of blood was collected via jugular venipuncture in sterile syringes. Samples were split into two tubes, processed as described above, and sent priority-overnight for CBC and serum chemistries to ANTECH Diagnostics (Fountain Valley, CA, USA). The same parameters as described previously were measured.

On the first day of dosing, 3 mL of blood was collected for a pharmacokinetic (PK) analysis from only 6 of the 8 dogs and cats in the study at each time point. Only the most cooperative dogs and cats were selected for the PK analysis. Blood was collected at 0, 1, 4, 8 and 24 h for cats and 0, 0.5, 1, 2, 4, 8 and 24 h for dogs to assess PK after treatment. Samples were placed into a redtop clotting tube. Serum was harvested by centrifuging the tubes at 1512× *g* for 15 min. The harvested serum was placed in cyrovials stored at −70 degrees Celsius. Samples were shipped overnight on dry ice to Proteomics and Metabolomics Facility at Colorado State University (Fort Collins, CO, USA).

### 2.3. Serum Cannabidiol (CBD) Extraction and Mass Spectrometry Analysis

CBD was extracted from canine and feline serum using a combination of protein precipitation and liquid–liquid extraction using n-hexane as previously described [19], with minor modifications for microflow ultra-high pressure liquid chromatography (UHPLC). Briefly, 0.05 mL of canine and feline serum was subjected to protein precipitation in the presence of ice-cold acetonitrile (200 μL; 80% final concentration in distilled water), spiked with deuterated CBD as the internal standard (0.06 mg/mL, CBD-d3 Cerilliant, Round Rock, TX, USA). 0.2 mL of water was added to each sample prior to the addition of 1 mL of hexane to enhance liquid–liquid phase separation. Hexane extract was removed and dried under laboratory nitrogen. Prior to liquid chromatography–mass spectrometry (LC–MS) analysis, samples were resuspended in 0.06 mL of 100% acetonitrile. A standard curve using the CBD analytical standard was prepared in canine and feline serum non-exposed to CBD and extracted as above. Cannabidiol concentration in serum was quantified using a chromatographically coupled triple-quadrupole mass spectrometer (UHPLC–QQQ-MS) using similar methods as previously described [20].

### 2.4. CBD Serum Concentration Data Analysis

From the UHPLC–QQQ-MS data, peak areas were extracted for CBD detected in biological samples and normalized to the peak area of the internal standard CBD-d3, in each sample using Skyline [21] as well as an in-house R Script (www.r-project.org). CBD concentrations were calculated to nanograms per mL of serum as determined by the line of regression of the standard curve (r^2^ = 0.9994, 0–1000 ng/mL). For this assay, the limits of detection (LOD) and limits of quantification (LOQ) represent the lower limits of detection and quantification for each compound in the matrix of this study [22,23].

### 2.5. Physical Examination

Physical evaluations were performed prior to the start of the study by the staff veterinarian and weekly thereafter for the duration of the trial. Qualified trained animal attendants performed adverse event observations twice daily for the duration of the study for signs of vomiting, loose stool, pain, or distress.

### 2.6. Data Analysis

Single-dose pharmacokinetics data is reported as area under the curve (AUC), maximum concentration (Cmax), time to maximal concentration (Tmax), elimination half-life (T ½), and mean retention time (MRT) (PK Solutions V.2, Montrose, CO, USA). Statistical analysis was performed with a commercially available software package (JMP 12.0, Cary, NC, USA). All continuous data were assessed utilizing a Shapiro–Wilk test for normality. Considering a majority of our blood and serum data were normally distributed a one-way analysis of variance was used to analyze these outcomes with Dunn’s post hoc testing for differences between baseline treatment and the other time points. A *p*-value 0.05 or less was deemed significant.

## 3. Results

### 3.1. Pharmacokinetics

All 6 dogs were dosed with soft chews at a dose of 2 mg/kg CBD/CBDA at 6 am in the morning. In all but one dog the entire dose was consumed, where it was realized the dog did not consume a portion of the dose at time 0 and was thus dropped form the experimental pharmacokinetic data set. Pharmacokinetics in dogs demonstrated that CBD T ½ life mean was 1 h for the 2 mg/kg dose (Table 1). Cmax of CBD was 301 ng/mL with a Tmax of 1.4 h MRT was 1.4 h and the mean AUC was 1297 ng-h/mL.

**Figure 1:**
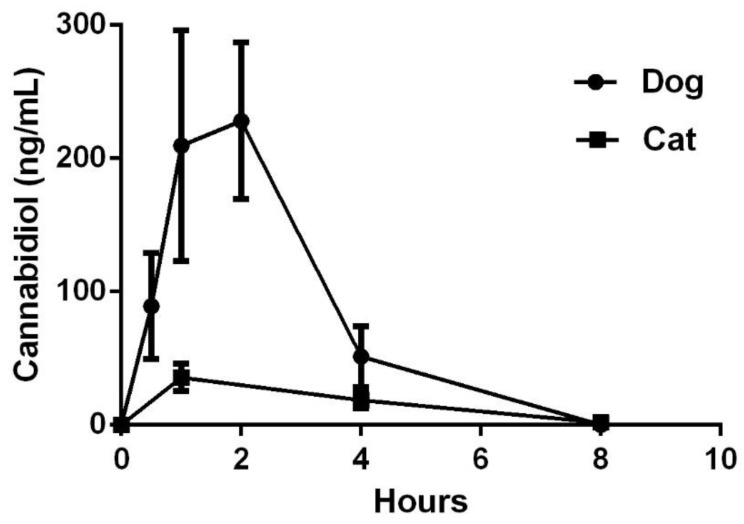
Mean and standard error of the mean (SEM) cannabidiol concentrations from dogs (*n* = 5) and cats (*n* = 6) at different time points after dosing.

All 8 cats were fasted overnight and dosed with fish oil filled capsules at 6:00 a.m. Two cats were observed to salivate heavily after administration and the study staff was not confident that two of the participants received the entire dose due to capsule rupture and were dropped from the pharmacokinetic study portion. Pharmacokinetics in the six compliant cats demonstrated the CBD T ½ life mean was 1.5 h for the 2 mg/kg dose (Table 2). The Cmax of CBD was 43 ng/mL with a Tmax of 2 h. MRT was only 3.5 h and the mean AUC was 164 ng-h/mL Mean and standard error of the mean (standard error of the mean (SEM)) concentrations from 0–8 h for dogs and cats are in Figure 1.

### 3.2. Complete Blood Counts (CBC) and Chemistry

CBC or serum biochemistry values outside of the reference ranges at any time point were not observed during the 12-week trail. (Table 3 and Table 4). CBC results for dogs show no alterations other than a small decrease in mean corpuscle volume with no changes in RBC morphology on examination at any time point.

On serum chemistry evaluations there were no statistically significant differences across the entire spectrum of chemistry values. ALP or ALT levels did not exceed the normal reference range during the study for any dog.

CBC results for the cats at all four time points are in Table 5. Cats exhibited no significant changes in mean cell counts over time, except for small significant decrease in eosinophil counts (*p* = 0.02).

**Figure 2:**
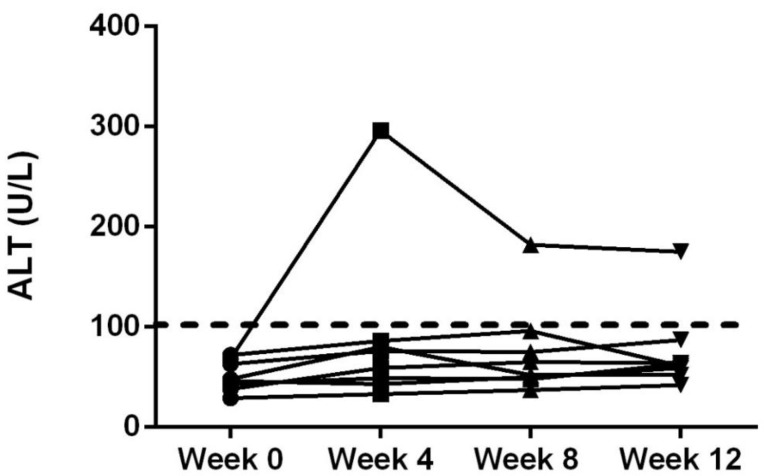
Serum alanine amino transferase (ALT) concentrations of all cats at time 0, 4, 8 and 12 weeks after oral dosing at 2 mg/kg twice daily. Dashed line represents the upper level of the reference range.

Serum biochemistry values were not observed to be outside of the normal ranges at any time point for the cats other than a single cat with elevated ALT level during treatment (Table 6; Figure 2). On serum chemistry evaluation there was a significant decrease in BUN over time, which was different from baseline values at week 8 and week 12 (*p* < 0.01). Serum triglycerides were also found to be decreased from baseline at week 8 and 12 (*p* = 0.02). Serum CK activity decreased over time being lower than baseline values at week 4, 8 and 12 (*p* < 0.01).

### 3.3. Physical Examination and Treatment Acceptance

Dogs were observed for signs of adverse events twice a day for the 12-week study. Out of 1344 total observation periods, 53 adverse events were reported. Loose stool was the most common adverse event noted among the eight dogs and occurred 44 times (3.3% of the time). Vomiting was recorded as either food or bile emesis. Vomiting was only recorded 6 times (0.45% of the time). For the duration of the study there was a high rate of acceptance of the CBD-infused chews, with an average acceptance rate of 96.7%. Five of the eight dogs had a 100% acceptance rate, on rare occasion 3 of the 8 dogs required manual treatment administration. Food consumption and body weight remained consistent during the 12 weeks. Physical examinations revealed no abnormalities or changes in behavior in the dogs throughout the study. The mean average weight change for the dogs during the 12 weeks of the study was −0.04 kg (−0.43%) which was not significant over the 12-week trial.

Cats were observed for signs of adverse events twice a day for the 12-week study for a total of 1344 observation periods. The main adverse effects noted included licking and head shaking, which were observed 476 (35.4%) and 339 (25.2%) times, respectively. Other adverse events noted were pacing (n = 150, 11.1%), chomping/chewing (n = 88, 6.5%), gagging (n = 29, 2.1%), vomiting food, bile, or hairballs (n = 15, 1.1%), salivating, drooling, or foaming (n = 16, 1.2%), jumping (n = 6, 0.45%), being uncooperative (n = 5, 0.4%), and grimacing (n = 5, 0.4%). Loose stool was not observed in any of the cats during the study. Food consumption and body weight of the cats remained consistent during the 12 weeks. Physical examinations revealed no abnormalities or changes in behavior in the cats throughout the study. The mean average weight change for the cats during the 12 weeks of the study was +0.06 kg (+1.04%).

## 4. Discussions

The serum pharmacokinetics of oral CBD-rich hemp nutraceuticals in dogs has been limited, with no data available in cats [11,12]. In dogs and cats respectively, the present pharmacokinetic study showed a mean Cmax of 301 ng/mL and 43 ng/mL, AUC of 1297 ng-h/mL and 36 164 ng-h/mL, and Tmax of 1.4 h and 2.0 h. Recent studies in dogs have shown that delivery of between 2–20 mg/kg of CBD in an oil base appears to be the preferred method of delivery for absorption, whereby oil beadlets and transdermal approaches are also effective, but not as effective as infused oils [11]. One aim of this study was to examine an infused soft chew treat made with a glycerol/starch/fiber base which should be easily digestible and appears to deliver approximately two and a half times the concentration as previously observed using an oil base [12], however the retention and half-life times appear to be shorter: between 1–2 h. The reasons for these differences are unclear. The prior study used small volumes of oil which might slowly transcend the esophagus into the stomach possibly prolonging the absorption; while the current delivery of a soft chew is more likely to create a food bolus that could be delivered to the stomach rapidly, thus allowing for quicker digestion and absorption. The fact that CBDA makes up half of the total CBD concentration in the chew may also be influencing this rapid and enhanced absorption since serum CBD concentrations tend to be higher when human participants are provided CBDA when compared to an equal amount of CBD [24].

Human data suggests that the oral absorption of CBD in fasting individuals is less than 10% of the dose. Recent pharmacokinetics in humans show that giving 1500 mg of CBD with food increases absorption 4–5 fold [25,26]. This appears to be true for other cannabinoids since primates given a THC-laden oatmeal cookie versus a sesame oil base in gelatin capsules display three-fold better absorption rates and five-fold higher Cmax concentrations [27]. In our opinion oral dosing with CBD in an oil base may enhance absorption, and may have been enhanced further in this study by inclusion in a food matrix. We have no explanation for our finding that elimination of CBD in the present work was significantly faster than in our previous study.

This brings into question the potential for therapeutic uses at our current dosing of 2 mg/kg twice a day (4 mg per day) and whether this may be enough to achieve therapeutic concentrations of CBD. The most current information from a canine seizure study suggests that therapeutic dosing might be approximately 2.5 mg/kg of CBD, and our positive clinical study in canine osteoarthritis using a whole plant extract showed benefits at 2 mg/kg [12,18]. This is in contrast to the current human dosing for children with specific forms of epilepsy which stands at 5–10 mg/kg twice daily of purified CBD [26,28,29]. In children with intractable epilepsy, when using a CBD-rich whole plant extract the effective dose can be lowered to approximately 2–3 mg/kg twice daily [29]. Further meta-analysis of whole-plant CBD-rich extract vs. synthetic CBD also indicates that lower doses are needed when using whole-plant extract [30]. When looking more closely at serum concentrations in the canine seizure study the effective serum concentrations of CBD are likely between 200–800 ng/mL, [11] however the methods for administering the product (i.e., with or without food) were not reported and might be the reason for the large variation in serum concentrations observed. Further work is needed to better understand how food influences serum absorption and steady-state pharmacokinetics of CBD which may help explain some of the variations in clinical studies. The differences in CBD serum concentrations in our pharmacokinetics and prior initial pilot study observations suggest there may be a role for clinically monitoring serum concentrations of CBD; if it were to be used by veterinarians [12,18].

In this uncontrolled preliminary study dosing of 2 mg/kg twice daily as an even mixture of CBD and CBDA showed no abnormalities in weekly physical examinations, nor any evidence of organ dysfunction as assessed by blood parameters. The canine CBD-infused chews showed no ALP elevations, with no ALP values falling outside the reference range (5–131 U/L) for any dog in the study. In a recent study, significant elevations in ALP values were noted when larger doses (10 and 20 mg/kg) were used in a 6-week trial [13]. When a clinical population of dogs was evaluated while utilizing a CBD-oil for the treatment of osteoarthritis pain at 2 mg/kg twice daily in dogs, 9 dogs exhibited rises in ALP with some being outside of the reference range in this primarily geriatric population [12]. Similarly, in a study using a different extract than the one used in this study at 2.5 mg/kg there were significant rises in ALP in many of the dogs’ undergoing seizure management [11]. The increases in ALP seen with CBD treatment are likely due to induction of cytochrome p450 mediated oxidative metabolism of the liver previously reported with prolonged exposure to cannabis [14,15]; however, in these clinical populations of dogs there were concomitant non-steroidal or seizure medications being given. The discrepancy between ALP rises in dogs with clinical diseases versus these healthy dogs receiving a similar dose is concerning and further studies are needed to better understand the hepatic response to cannabinoids.

During this preliminary investigation, 97% acceptance of the soft chew was observed. The only side effects noted were occasional episodes of loose stool and vomiting observed in the dogs (3.3% of total observations). This is well within the typical occurrence at this specific contract laboratory where the incidence of diarrhea is approximately 3%.

To date, the absorption of CBD in cats from an oral preparation has not been studied. On initiation of the CBD-infused fish oil product two of the cats given the dose in the encapsulated form showed salivation and head shaking due to broken capsules during administration, therefore only 6 of the 8 cats had pharmacokinetic testing. Overall, the absorption kinetics showed maximal serum concentration are approximately one-fifth of what was observed in the dogs (mean Cmax of 43 ng/mL) with a longer retention time at 3.5 h and a half-life of 2.4 h. It is entirely possible that we missed the peak Cmax since a 2 h time point was not collected in this cohort due to blood collection times being limited by the physical size of cats, but if absorption is similar to dogs the serum concentrations would have been similar at 2 h [11,13]. These findings suggest that the absorption of CBD in the fish oil base is less than in dogs using plant oil bases, hence larger doses may be necessary for pharmacological effects. This information is important for feline practitioners that are considering the use of CBD products in cats for anxiety, arthritis, house soiling, seizure activity, or neoplasia, which are all reported as maladies where owners are using CBD products [1].

When examining the complete blood count and the serum chemistry results, there were some changes in a few parameters for cats. The feline CBD-infused fish oil did not alter ALT in all but one cat. One cat showed maintained elevated ALT changes above the upper reference-range limits throughout the study after initiation of treatment. No clinical signs were observed that could be directly linked to the increased ALT. This rise could be due to hepatocellular injury because of an unknown disease process in the one cat or due to the CBD-infused oil. In addition, there were small decreases in triglyceride, creatine kinase and blood urea nitrogen, which may be attributed to the CBD or the fish-oil treatment. The lack of controls during this study does not allow for speculation regarding the effects of fish oil or CBD, as both have been associated with alterations in triglyceride and creatine kinase [31,32,33,34]. Further long-term dose escalations studies are warranted examining the serum chemistry changes with particular attention paid to the hepatic enzymes.

During the study, some of the cats were observed to have negative effects associated with oil administration. Considering the lack of a control group receiving fish oil alone we cannot say whether this reaction was due to fish oil or the hemp-volatile molecules. For these reasons, other delivery methods examining pharmacokinetics and toxicity should be considered, such as transdermal or transfollicular administration.

Besides the lack of a control group as a major limitation discussed above, other limitations must be recognized. This was a small homogenous population of dogs and cats being utilized in a contract research facility which may not reflect the companion animal dog or cat living in a common household. Additionally, it does not reflect what is occurring in dogs or cats with comorbidities that are being treated with other pharmaceuticals in an aged population. The use of this particular product cannot be used to determine the global safety of all products since many of these hemp products are not pure CBD products and have other cannabinoids and terpenes in smaller quantities that may affect cats and dogs differently [35]. Lastly, due to logistics regarding sample collection and shipping, we did not get steady-state concentrations after 12 weeks of administration, therefore we cannot comment on serum concentrations after chronic exposure in either species. Overall, the lack of negative CBC and serum chemistry findings in a modest duration toxicity/tolerance trial of this nature are encouraging regarding the safe use of this specific dose and this specific CBD-rich hemp product.

In conclusion, hemp-based CBD appears to be relatively safe in healthy populations of dogs and cats, and dogs appear to absorb CBD better than cats. The lack of serum chemistry alterations in both species is comforting as it relates to preliminary toxicity findings; however, use of CBD-rich hemp products requires monitoring of liver enzyme values. Continued clinical follow up is essential in those patients undergoing long-term use with naturally occurring disease who may be on other treatments for their ailments. Further studies are warranted to determine safety in dogs and cats. Studies should also examine drug interactions in patients on multiple medications, especially in those that are highly metabolized by the hepatic cytochrome p450 system.

## Figures and Tables

**Table 1:** Single dose serum pharmacokinetics of 2 mg/kg oral dose of cannabidiol (CBD) enriched soft chew of individual dogs with mean and standard error.

Dog	Cmax (ng/mL)	Tmax (h)	T1/2 el. (h)	AUC 0-t (ng-h/mL)	MRT (h)
1	266	2	1.8	1494	2.6
2	315	1	0.5	1431	0.7
3	242	1	0.8	803	1.1
4	151	2	1.1	845	1.6
5	531	1	0.8	1912	1.2
Mean ± SEM	301 ± 63	1.4 ± 0.2	1.0 ± 0.2	1297 ± 210	1.4 ± 0.3

Cmax = maximum concentration; Tmax = time of maximum concentration; T1/2 el = half-life of elimination; AUC 0-t = area under the curve (time 0 to 24 h); MRT = median residence time.

**Table 2:** Single dose serum pharmacokinetics of 2 mg/kg oral dosage of CBD-infused fish oil of individual cats with mean and standard error.

Cat	Cmax (ng/mL)	Tmax (h)	T1/2 elim (h)	AUC 0-t (ng-h/mL)	MRT (h)
1	75	1	1.2	212	2.1
2	41	1	1.3	125	2.4
3	53	1	1.7	194	2.9
4	21	4	1.7	134	5.4
5	20	1	1.7	60	2.7
6	48	4	1.2	256	5.7
Mean + SE	43 ± 9	2.0 ± 0.6	1.5 ± 0.1	164 ± 29	3.5 ± 1.4

Cmax = maximum concentration; Tmax = time of maximum concentration; T1/2 el = half-life of elimination; AUC 0-t = area under the curve (time 0 to 24 h); MRT = median residence time.

**Table 3:** Dog (*n* = 8) complete blood count means and SEM immediately prior to (week 0), 4 weeks, 8 weeks and 12 weeks of an oral 2 mg/kg CBD dose twice daily using a CBD-rich hemp product.

Comp. Blood Count (Ref. Range) **	Week 0	Week 4	Week 8	Week 12	*p*-Value
WBC (4.0–15.5 × 10^3^/mm^3^)	8.4 ± 0.7	7.7 ± 0.5	7.3 ± 1	7.7 ± 0.7	0.22
RBC (4.8–9.3 × 10^6^/mm^3^)	7.5 ± 0.3	7.3 ± 0.2	7.9 ± 0.1	7.5 ± 0.1	0.35
Hb (12.1–20.3 g/dL)	17.5 ± 0.6	17.1 ± 0.5	17.9 ± 0.2	17.8 ± 0.3	0.32
Hct (36–60%)	54 ± 1	53 ± 1	57 ± 2	51 ± 1	0.73
MCV (58–79 μm^3^)	73 ± 1	72 ± 2	73 ± 1	69 ± 1 *	<0.01
MCH (19–28 μg)	24 ± 0	23 ± 1	23 ± 0	24 ± 0	0.92
MCHC (30–38 g/dL)	33 ± 1	33 ± 1	32 ± 1	35 ± 1	0.34
Platelets (170–400 × 10^3^/mm^3^)	318 ± 18	311 ± 15	304 ± 11	347 ± 19	0.16
Neutrophils (2060–10,600/μL)	5508 ± 567	5216 ± 388	4889 ± 740	5221 ± 586	0.64
Lymphocytes (690–4500/μL)	2347 ± 59	1912 ± 65	1960 ± 131	1904 ± 160	0.07
Monocytes (0–840/μL)	361 ± 20	297 ± 36	335 ± 66	359 ± 70	0.88
Eosinophils (0–1200/μL)	198 ± 11	238 ± 36	154 ± 23	181 ± 22	0.28

* Represents a parameter that was significantly (*p* < 0.05) different from baseline. ** Klaassen, J.K. Reference values in veterinary medicine. Lab Med, 1999, 30, 194–197.

**Table 4:** Dog (*n* = 8) mean and SEM of serum chemistry parameters immediately prior to (week 0), 4 weeks, 8 weeks and 12 weeks of an oral 2 mg/kg CBD dose twice daily using a CBD-rich hemp product.

Serum Chemistry (Ref Range) **	Week 0	Week 4	Week 8	Week 12	*p*-Value
TP (5.0–7.4 g/dL)	6.1 ± 0.1	5.9 ± 0.2	6.3 ± 0.2	6.0 ± 0.2	0.65
Albumin (2.7–4.4 g/dL)	3.5 ± 0.1	3.5 ± 0.1	3.5 ± 0.1	3.4 ± 0.1	0.22
Globulin (1.6–3.6 g/dL)	2.6 ± 0.1	2.5 ± 0.1	2.9 ± 0.1	2.6 ± 0.2	0.18
AST (15–66 U/L)	27 ± 2	25 ± 2	23 ± 2	25 ± 1	0.45
ALT (12–118 U/L)	34 ± 3	27 ± 2	35 ± 10	28 ± 3	0.57
ALP (5–131 U/L)	39 ± 6	46 ± 7	56 ± 10	61 ± 13	0.09
GGT (1–12 U/L)	4 ± 0	3 ± 0	4 ± 0	4 ± 0	0.72
BUN (6–31 mg/dL)	11 ± 1	10 ± 1	11 ± 1	11 ± 0	0.82
Creatinine (0.5–1.6 mg/dL)	0.5 ± 0.0	0.5 ± 0.0	0.5 ± 0.0	0.5 ± 0.0	0.36
Phosphorous (2.5–6.0 mg/dL)	4.3 ± 0.2	4.1 ± 0.2	4.2 ± 0.3	4.0 ± 0.2	0.11
Glucose (70–138 mg/dL)	97 ± 3	92 ± 2	102 ± 3	99 ± 2	0.16
Calcium (8.9–11.4 mg/dL)	10.4 ± 0.1	10.0 ± 0.1	10.2 ± 0.1	10.1 ± 0.1	0.16
Magnesium (1.5–2.5 mEq/L)	1.6 ± 0.0	1.6 ± 0.0	1.6 ± 0.0	1.6 ± 0.0	0.11
Sodium (139–154 mEq/L)	148 ± 0	148 ± 0	146 ± 1	148 ± 0	0.58
Potassium (3.6–5.5 mEq/L)	4.3 ± 0.1	4.4 ± 0.1	4.3 ± 0.1	4.2 ± 0.0	0.23
Chloride (102–120 mEq/L)	113 ± 0	113 ± 1	111 ± 1	113 ± 1	0.74
Cholesterol (92–324 mg/dL)	182 ± 13	203 ± 12	211 ± 12	212 ± 17	0.06
Triglycerides (29–291 mg/dL)	48 ± 4	44 ± 4	43 ± 5	46 ± 6	0.44
Creatine Kinase (59–895 U/L)	130 ± 16	142 ± 43	83 ± 5	97 ± 5	0.10

** Klaassen, J.K. Reference values in veterinary medicine. Lab Med, 1999, 30, 194–197.

**Table 5:** Cat (n = 8) mean and SEM of complete blood counts immediately prior to (week 0), 4 weeks, 8 weeks and 12 weeks of an oral 2 mg/kg CBD dose twice daily using a CBD-rich hemp product.

Comp. Blood Count (Ref. Range) **	Week 0	Week 4	Week 8	Week 12	*p*-Value
WBC (3.5–16.0 × 10^3^/μL)	14.0 ± 1.6	13.6 ± 1.5	12.9 ± 1.3	12.5 ± 1.6	0.10
RBC (5.9–15.9 × 10^6^/μL)	8.8 ± 0.2	8.0 ± 0.3	9.0 ± 0.2	9.0 ± 0.3	0.22
Hb (9.3–15.9 g/dL)	11.4 ± 0.4	10.7 ± 0.4	12.4 ± 0.4	11.8 ± 0.5	0.06
Hct (29–48%)	39 ± 1	34 ± 1	40 ± 1	39 ± 2	0.48
MCV (37–61 fL)	44 ± 1	42 ± 1	44 ±1	43 ± 1	0.86
MCH (11–21 pg)	13 ± 0	13 ± 1	13 ± 1	13 ± 1	0.16
MCHC (30–38 g/dL)	30 ± 2	32 ± 0	31 ± 1	31 ± 0	0.12
Platelets (200–500 × 10^3^/μL)	333 ± 31	374 ± 30	361 ± 20	289 ± 19	0.08
Neutrophils (2500–8500/μL)	7980 ± 1081	8993 ± 1124	7394 ± 1082	7847 ± 1349	0.70
Lymphocytes (1200–8000/μL)	4481 ± 518	3314 ± 782	3856 ± 803	3614 ± 1052	0.24
Monocytes (0–600/μL)	428 ± 138	416 ± 66	545 ± 36	364 ± 73	0.08
Eosinophils (0–1000/μL)	1149 ± 148	876 ± 139	1026 ± 212	650 ± 91 *	0.02

* Represents a parameter that was significantly (*p* < 0.05) different from baseline. ** Klaassen, J.K. Reference values in veterinary medicine. Lab Med, 1999, 30, 194–197.

**Table 6:** Cat (n = 8) mean and SEM of serum chemistry parameters immediately prior to (week 0), 4 weeks, 8 weeks and 12 weeks of an oral 2 mg/kg CBD dose twice daily using a CBD-rich hemp product.

Serum Chemistry (Ref. Range) **	Week 0	Week 4	Week 8	Week 12	*p*-Value
TP (5.2–8.8 g/dL)	7.2 ± 0.2	6.7 ± 0.2	7.1 ± 0.2	7.1 ± 0.2	0.94
Albumin (2.5–3.9 g/dL)	3.2 ± 0.1	3.2 ± 0.1	3.4 ± 0.1	3.2 ± 0.1	0.65
Globulin (2.3–5.3 g/dL)	4.0 ± 0.2	3.5 ± 0.2	3.8 ± 0.2	3.9 ± 0.2	0.72
AST (10–100 U/L)	21 ± 2	24 ± 4	24 ± 3	24 ± 3	0.17
ALT (10–100 U/L)	51 ± 5	90 ± 30	76 ± 17	75 ± 15	0.29
ALP (6–102 U/L)	30 ± 5	30 ± 6	29 ± 5	28 ± 6	0.53
GGT (1–10 U/L)	1 ± 0	1 ± 0	2 ± 0	1 ± 0	0.91
BUN (14–36 mg/dL)	23 ± 1	22 ± 1	20 ± 1 *	19 ± 1 *	<0.01
Creatinine (0.6–2.4 mg/dL)	1.3 ± 0.1	1.3 ± 0.0	1.3 ± 0.1	1.3 ± 0.1	0.84
Phosphorous (2.4–8.2 mg/dL)	4.5 ± 0.4	4.6 ± 0.4	4.3 ± 0.4	4.1 ± 0.2	0.06
Glucose (64–170 mg/dL)	90 ± 2	85 ± 2	88 ± 3	86 ± 3	0.25
Calcium (8.2–10.8 mg/dL)	9.6 ± 0.1	9.0 ± 0.1	9.5 ± 0.2	9.3 ± 0.1	0.72
Magnesium (1.5–2.5 mEq/L)	1.9 ± 0.1	1.8 ± 0.0	1.8 ± 0.0	1.8 ± 0.0	0.65
Sodium (145–158 mEq/L)	151 ± 1	153 ± 1	154 ± 1	151 ± 1	0.39
Potassium (3.4–5.6 mEq/L)	4.7 ± 0.2	4.7 ± 0.2	4.7 ± 0.3	4.5 ± 0.1	0.30
Chloride (104–128 mEq/L)	119 ± 1	121 ± 1	122 ± 1	119 ± 1	0.33
Cholesterol (75–220 mg/dL)	139 ± 9	123 ± 6	128 ± 6	123 ± 7	0.09
Triglycerides (25–160 mg/dL)	32 ± 1	28 ± 2	26 ± 2 *	25 ± 2 *	0.02
Creatine Kinase (59–529 U/L)	197 ± 31	113 ± 15 *	106 ± 12 *	126 ± 14 *	<0.01

* Represents a parameter that was significantly (*p* < 0.05) different from baseline. ** Klaassen, J.K. Reference values in veterinary medicine. Lab Med, 1999, 30, 194–197.

## References

[1] Kogan L.R., Hellyer P.W., Robinson N.G. (2017). Consumers perception fo hemp products for animals. J. Am. Holist. Vet. Med. Assoc..

[2] Landa L., Sulcova A., Gbelec P. (2016). The use of cannabinoids in animals and therapeutic implications for veterinary medicine: A review. Vet. Med..

[3] Pisanti S., Malfitano A.M., Ciaglia E., Lamberti A., Ranieri R., Cuomo G., Abate M., Faggiana G., Proto M.C., Fiore D. (2017). Cannabidiol: State of the art and new challenges for therapeutic applications. Pharm. Ther..

[4] Fraguas-Sánchez A.I., Torres-Suárez A.I. (2018). Medical Use of Cannabinoids. Drugs.

[5] Zhornitsky S., Potvin S. (2012). Cannabidiol in humans—The quest for therapeutic targets. Pharmaceuticals.

[6] Yamamoto I., Watanabe K., Narimatsu S., Yoshimura H. (1995). Recent advances in the metabolism of cannabinoids. Int. J. Biochem. Cell Biol..

[7] Brutlag A., Hommerding H. (2018). Toxicology of Marijuana, Synthetic Cannabinoids, and Cannabidiol in Dogs and Cats. Vet. Clin. Small Anim..

[8] Bergamaschi M.M., Costa-Querioz R.H., Crippa J.A.S., Zuardi A.W. (2011). Safety and Side Effects of Cannabidiol, a Cannabis sativa Constituent. Curr. Drug Saf..

[9] Russo E.B. (2019). The Case for the Entourage Effect and Conventional Breeding of Clinical Cannabis: No Strain, No Gain. Front. Plant Sci..

[10] Ruhaak L.R., Felth J., Karlsson P.C., Rafter J.J., Verpoorte R., Bohlin L. (2011). Evaluation of the cyclooxygenase inhibiting effects of six major cannabinoids isolated from Cannabis sativa. Biol. Pharm. Bull..

[11] McGrath S., Bartner L.R., Rao S., Packer R.A., Gustafson D.L. (2019). Randomized blinded controlled clinical trial to assess the effect of oral cannabidiol administration in addition to conventional antiepileptic treatment on seizure frequency in dogs with intractable idiopathic epilepsy. J. Am. Vet. Med. Assoc..

[12] Gamble L.J., Boesch J.M., Frye C.W., Schwark W.S., Mann S., Wolfe L., Brown H., Berthelsen E.S., Wakshlag J.J. (2018). Pharmacokinetics, safety, and clinical efficacy of cannabidiol treatment in osteoarthritic dogs. Front. Vet. Sci..

[13] McGrath S., Bartner L.R., Rao S., Kogan L.R., Hellyer P.W. (2018). A report of adverse effects associated with the administration of cannabidiol in healthy dogs. J. Am. Holist. Vet. Med. Assoc..

[14] Jiang R., Yamaori S., Takeda S., Yamamoto I., Wantanabe K. (2011). Identification of cytochrome P450 enzymes responsible for metabolism of cannabidiol by human liver microsomes. Life Sci..

[15] Bornheim L.M., Correia M.A. (1989). Effect of cannabidiol on cytochrome P-450 isozymes. Biochem. Pharm..

[16] Harvey D.J., Samara E., Mechoulam R. (1991). Comparative metabolism of cannabidiol in dog, rat and man. Pharmacol. Biochem. Behav..

[17] Samara E., Bialer M., Harvey D.J. (1990). Pharmacokinetics of urinary metabolites of cannabidiol in the dog. Biopharm. Drug Dispos..

[18] Bartner L.R., McGrath S., Rao S., Hyatt L.K., Wittenburg L.A. (2018). Pharmacokinetics of cannabidiol administered by 3 delivery methods at 2 different dosages to healthy dogs. Can. J. Vet. Res..

[19] Zgair A., Wong J.C.M., Sabri A., Fischer P.M., Barrett D.A., Constantinescu C.S., Gershkovich P. (2015). Development of a simple and sensitive HPLC-UV method for the simultaneous determination of cannabidiol and Δ(9)-tetrahydrocannabinol in rat plasma. J. Pharm. Biomed. Anal..

[20] Kirkwood J.S., Broeckling C.D., Donahue S., Prenni J.E. (2016). A novel microflow LC-MS method for the quantitation of endocannabinoids in serum. J. Chromatogr. B Anal. Technol. Biomed. Life Sci..

[21] MacLean B., Tomazela D.M., Shulman N., Chambers M., Finney G.L., Frewen B. (2010). Skyline: An open source document editor for creating and analyzing targeted proteomics experiments. Bioinformatics.

[22] Broccardo C.J., Schauer K.L., Kohrt W.M., Schwartz R.S., Murphy J.P., Prenni J.E. (2013). Multiplexed analysis of steroid hormones in human serum using novel microflow tile technology and LC–MS/MS. J. Chromatogr. B Anal. Technol. Biomed. Life Sci..

[23] Shrivastava A., Gupta V.B. (2011). Methods for the determination of limit of detection and limit of quantitation of the analytical methods. Chron. Young Sci..

[24] Eichler M., Spinedi L., Unfer-Grauwiler S., Bodmer M., Surber C., Luedi M., Drewe J. (2012). Heat exposure of Cannabis sativa extracts affects the pharmacokinetic and metabolic profile in healthy male subjects. Planta Med..

[25] Millar S.A., Stone N.L., Yates A.S., O’Sullivan S.E. (2018). A Systematic Review on the Pharmacokinetics of Cannabidiol in Humans. Front. Pharmacol..

[26] Taylor L., Gidal B., Blakey G., Tayo B., Morrison G. (2018). A Phase I, Randomized, Double-Blind, Placebo-Controlled, Single Ascending Dose, Multiple Dose, and Food Effect Trial of the Safety, Tolerability and Pharmacokinetics of Highly Purified Cannabidiol in Healthy Subjects. CNS Drugs.

[27] Perlin E., Smith C.G., Nichols A.I., Almirez R., Flora K.P., Cradock J.C., Peck C.C. (1985). Disposition and bioavailability of various formulation of tetrahydrocannabinol in the Rhesus Monkey. J. Pharm. Sci..

[28] Huntsman R.J., Tang-Wai R., Alcorn J., Vuong S., Acton B., Corley S., Laprairie R., Lyon A.W., Meier S., Mousseau D.D. (2019). Dosage Related Efficacy and Tolerability of Cannabidiol in Children with Treatment-Resistant Epileptic Encephalopathy: Preliminary Results of the CARE-E Study. Front. Neurol..

[29] Birnbaum A.K., Karanam A., Marino S.E., Barkley C.M., Remmel R.P., Roslawski M., Gramling-Aden M., Leppik I.E. (2019). Food effect on pharmacokinetics of cannabidiol oral capsules in adult patients with refractory epilepsy. Epilepsia.

[30] Pamplona F.A., Da Silva L.R., Coan A.C. (2018). Potential Clinical Benefits of CBD-Rich Cannabis Extracts Over Purified CBD in Treatment-Resistant Epilepsy: Observational Data Meta-analysis. Front. Neurol..

[31] De Godoy M.R.C., McLeod K.R., Harmon D.L. (2018). Influence of feeding a fish oil-containing diet to mature, overweight dogs: Effects on lipid metabolites, postprandial glycaemia and body weight. J. Anim. Physiol. Anim. Nutr..

[32] Silvestri C., Paris D., Martella A., Melck D., Guadagnino I., Cawthorne M., Motta A., Di Marzo V. (2015). Two non-psychoactive cannabinoids reduce intracellular lipid levels and inhibit hepatosteatosis. J. Hepatol..

[33] Iannotti F.A., Pagano E., Moriello A.S., Alvino F.G., Sorrentino N.C., D’Orsi L., Gazzerro E., Capasso R., De Leonibus E., De Petrocellis L. (2018). Effects of non-euphoric plant cannabinoids on muscle quality and performance of dystrophic mdx mice. Br. J. Pharmacol..

[34] Marques C.G., Santos V.C., Levada-Pires A.C., Jacintho T.M., Gorjão R., Pithon-Curi T.C., Cury-Boaventura M.F. (2015). Effects of DHA-rich fish oil supplementation on the lipid profile, markers of muscle damage, and neutrophil function in wheelchair basketball athletes before and after acute exercise. Appl. Physiol. Nutr. Metab..

[35] Nie B., Henion J., Wakshlag J. (2019). Analysis of Veterinary Hemp-Based Oils for Product Integrity by LC/MS. Cannabis Sci. Technol..

